# Polaritonic Spectra of Optical Mie Voids

**DOI:** 10.1002/nap2.70097

**Published:** 2026-04-23

**Authors:** Evgeny Ryabkov, Mingzhao Song, Andrey A. Bogdanov, Denis G. Baranov

**Affiliations:** ^1^ Qingdao Innovation and Development Center of Harbin Engineering University Qingdao Shandong China; ^2^ Moscow Center for Advanced Studies Moscow Russia; ^3^ School of Physics and Engineering ITMO University Saint Petersburg Russia

**Keywords:** Mie resonances, Mie voids, polaritons, strong coupling

## Abstract

The progress in understanding the optical and microscopic properties of polaritons relies on various optical cavities to confine electromagnetic radiation, which causes a demand for new platforms with higher Q‐factors and better fabrication robustness. In this context, so called Mie voids—spherical cavities inside a dielectric medium, where the light confinement occurs due to refractive index contrast at the air‐dielectric interface—present a substantial interest. Here, we theoretically study the resonant characteristics and polaritonic spectra of spherical Mie cavities loaded with resonant media, as well as address the inverted problem, where a Mie void is formed inside a resonant dispersive medium. We establish approximate analytical expressions for the Q‐factors of Mie void cavities, find the parameter ranges of spherical voids leading to the regimes of weak and strong light–matter coupling, and analyze the concomitant effects, such as Q‐factor enhancement and spatial field localization, from the polaritonic perspective. Our result could be valuable for the design of new polaritonic systems.

## Graphical abstract

Optical Mie voids have been shown to provide a versatile platform for polaritonic physics. Analytical and numerical analysis reveal how void resonances couple to excitonic media, enabling weak‐ and strong‐coupling regimes, polariton gaps, Q‐factor enhancement, and spatial mode localization. The results establish Mie voids as a versatile platform for polaritonic and nanophotonic engineering in high‐permittivity dielectric cavities.

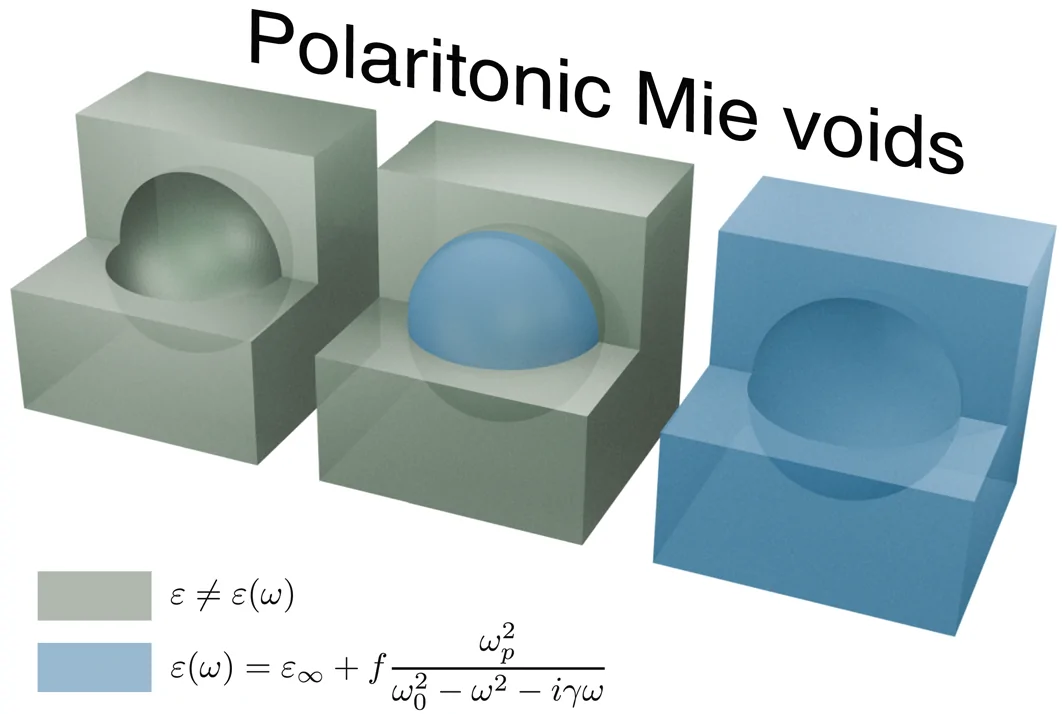

## 1 Introduction

Polaritons are hybrid eigenstates of an optical cavity interacting with material excitations—such as an electronic or a vibrational transition—in the nonperturbative regime [1, 2]. Thanks to their hybrid composition, polaritons offer opportunities to modify various microscopic properties of matter [3, 4], and even to control the rates of certain chemical reactions by altering molecular energy levels [5, 6, 7, 8, 9, 10]. The study of polaritons in the optical and infrared domains relies on various optical cavities to confine electromagnetic radiation. The progress in this field causes a steady demand for new platforms with higher Q‐factors, easier fabrication requirements, and better fabrication robustness [11].

Frequently used types of optical cavities incorporated in polaritonic systems include traditional Fabry–Pérot (FP) cavities [12, 13, 14], photonic crystal cavities [15], propagating surface modes [16, 17], individual plasmonic nanocavities [18, 19, 20, 21], and lattice resonances [22]. More recent research efforts resort to bulk [23] or so‐called cavity‐free polaritons [24, 25, 26, 27], where the confinement of electromagnetic radiation is facilitated by the same material, which hosts the material excitation itself [28].

In this context, voids inside a dielectric medium, where light confinement occurs due to the refractive index contrast at the air–dielectric interface, are of substantial interest. Depending on their geometry and dielectric properties of the host materials, such cavities were theoretically and experimentally proven to be a versatile platform for scattering control [29, 30, 31, 32] and absorption enhancement [33], as well as general resonance tailoring [34, 35]. Recently, spherical void resonators embedded in a dielectric medium have been reintroduced by Hentschel et al. [36] as dielectric Mie voids, because their modes can be described by the original Mie theory [37]. In a way, one can think of these optical modes as Mie resonances “inside‐out” [38, 39, 40]. Structures supporting Mie void resonances recently found elegant applications for molecular sensing [41], plastic microparticles sorting [42], as well as emission enhancement [43].

From the fundamental perspective, resonances of Mie voids correspond to regular quasi‐normal modes (QNMs) of an open photonic cavity embedded in a transparent dielectric medium [44, 45, 46, 47, 48]. A recent work [49] established an even deeper connection between Mie void resonances and Mie resonances of a dielectric sphere using the Babinet principle for a dielectric void.

Despite the extensive theoretical examination and utilization of Mie voids, they have not yet been considered as platforms for realizing strong light–matter coupling. Polaritonic solutions in the inverted cases—dielectric spheres incorporating resonant media—have been extensively studied in recent works as self‐hybridized polaritons [26, 50, 51]. In this regard, the question of realizing and analyzing polaritonic modes in Mie void cavities naturally arises.

In this paper, we theoretically study the resonant characteristics and polaritonic spectra of spherical cavities loaded (filled) with a generic resonant medium. We begin with the case of an empty Mie void (spherical cavity) surrounded by a nondispersive transparent dielectric medium to establish the dependencies of the QNMs on the size and background permittivities. Next, we examine the polaritonic spectra of these voids loaded with a resonant medium and analyze the coupling of the void quasi‐normal modes with the medium resonances. Within this picture, we establish the role of radial mode number and dielectric characteristics on the regime of coupling. Finally, we analyze the inverted problem where an empty Mie void is surrounded by a dispersive dielectric medium described by a Lorentz resonant transition. In this problem, we reveal the role of the background dielectric characteristics, mainly losses, on the resulting quality factors of the system and the regime of light–matter coupling. We also identify the regime of spatial QNM localization that occurs due to large absorption losses of the background medium close to the resonance.

## 2 Theoretical Background

### 2.1 Model and Main Equations

The system studied throughout the paper is represented by a spherical cavity (void) of radius R with permittivity $\varepsilon_{v}=n_{v}^{2}$ surrounded by a background medium $\varepsilon_{bg}=n_{bg}^{2}$. Both permittivities may be frequency‐dependent. Figure 1 illustrates three variations of the basic system that we analyze in the following: (a) an empty spherical void surrounded by a transparent dielectric background, (b) a dielectric‐surrounded spherical void loaded with a resonant medium, and (c) an empty spherical void embedded in a dispersive resonant medium. This last system establishes a connection to and explains certain spectral features observed earlier by Hentschel et al. [36] from the general polaritonic perspective.

**FIGURE 1:**
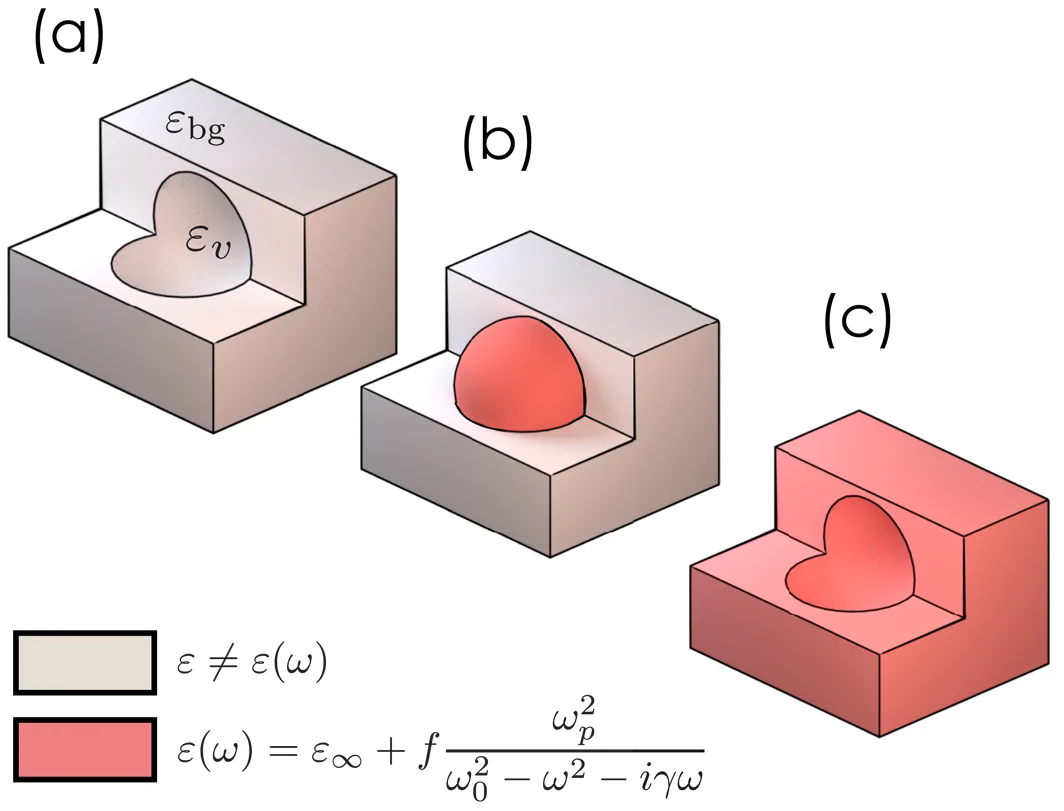
Schematics of the three classes of systems analyzed in this paper. (a) An empty Mie void inside a transparent dielectric; (b) a Mie void loaded with a resonant medium embedded in a transparent dielectric; (c) an empty Mie void embedded in a dispersive resonant medium. The resonant media are represented by the Lorentz model, Equation (5).

The resonant characteristics and polaritonic spectra of Mie voids realized with various materials are encoded in the optical eigenfrequencies of the structures. For a spherical cavity, the electromagnetic field of every QNM is represented by a single vector spherical harmonic with a particular polarization state (TE and TM), angular momentum ℓ, and magnetic number (projection of the orbital momentum) m. For any given polarization state and angular momentum ℓ, complex‐valued eigenfrequencies of the spherical cavity are given by the roots of the characteristic equation independent of m [37].

(1) $$\begin{array}{c} TM:\;n_{bg}\xi_{\ell }\left(n_{bg}kR\right)\psi_{\ell }^{\prime }\left(n_{v}kR\right)-\\ n_{v}\psi_{\ell }\left(n_{v}kR\right)\xi_{\ell }^{\prime }\left(n_{bg}kR\right)=0, \end{array}$$

(2) $$\begin{array}{c} TE:\;n_{v}\xi_{\ell }\left(n_{bg}kR\right)\psi_{\ell }^{\prime }\left(n_{v}kR\right)-\\ n_{bg}\psi_{\ell }\left(n_{v}kR\right)\xi_{\ell }^{\prime }\left(n_{bg}kR\right)=0 \end{array}$$

Here, k=ω/c, $\psi_{\ell }\left(x\right)=xj_{\ell }\left(x\right)$ and $\xi_{\ell }\left(x\right)=xh_{\ell }^{\left(1\right)}\left(x\right)$ are Riccati–Bessel functions, and $j_{\ell }\left(x\right)$ and $h_{\ell }^{\left(1\right)}\left(x\right)$ are spherical Bessel and Hankel functions of the first kind, respectively. The prime denotes derivatives with respect to the argument.

### 2.2 Eigenmode Structure of Mie Voids

Figure 2 shows the complex eigenfrequencies of TM and TE modes for ℓ=1,2,3, and 7 for a spherical Mie void of radius R=500nm, taken as an example. The dielectric background has a permittivity $\varepsilon_{\text{bg}}=9$, whereas the void is filled with air ($\varepsilon_{v}=1$). For each ℓ and polarization, the solutions of Equations (1) and (2) yield countably many complex‐valued eigenfrequencies, which we label with an index N=1,2,3,… that enumerates the roots of the characteristic equation. Although N is not a conserved quantum number, it provides a convenient ordering of the modes and reflects the increasing number of radial field oscillations. All solutions in a given series exhibit identical angular but different radial distributions; therefore, N can be associated with the radial quantum number. As seen in Figure 2, the spectrum naturally clustered into two groups with markedly different physical properties for each polarization and angular momentum.

**FIGURE 2:**
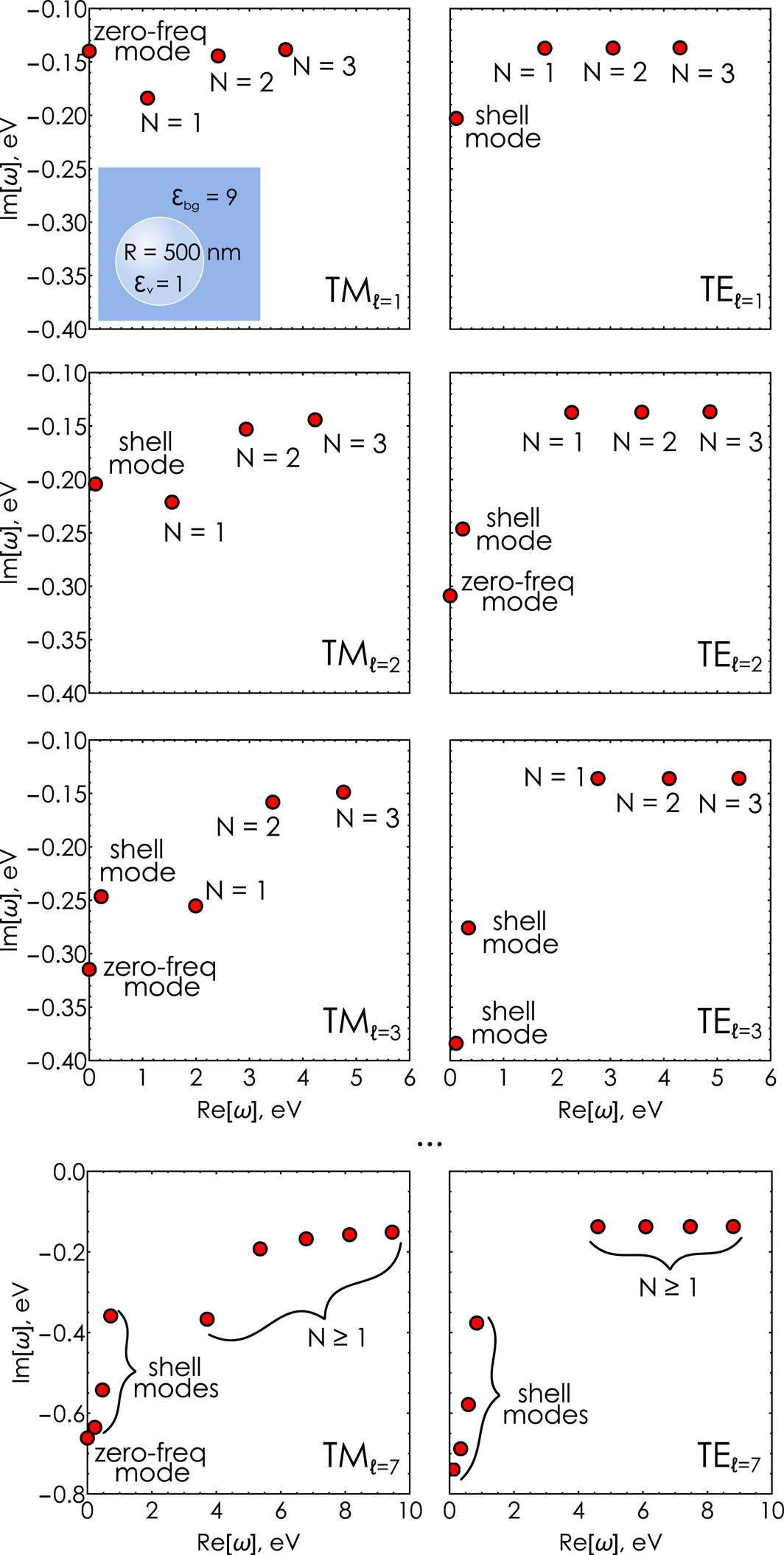
Complex frequencies of ${\mathrm{TM}}_{\ell =1,2,3,7}$ and ${\mathrm{TE}}_{\ell =1,2,3,7}$ quasinormal modes of an empty spherical Mie void (R=500 nm) in a transparent background ($\varepsilon_{bg}=9$) calculated as the roots of Equations (1) and (2), respectively. The QNMs are represented by a single zero‐frequency mode present for some combinations of polarization and angular momentum, a finite set of low‐Q shell modes, and an infinite set of high‐Q core modes.

The first group consists of modes with relatively small imaginary parts of the eigenfrequency. These modes, referred here to as *core modes* and indexed with the radial number N=1,2,…, are predominantly confined inside the cavity due to the dielectric contrast and correspond to the conventional Mie resonances of the cavity. They behave similarly to Fabry–Perot resonances in the complex plane and form an almost equidistant series of frequencies with nearly identical imaginary parts determined by the dielectric contrast $n_{\text{bg}}/n_{v}$. A more detailed analysis is presented in Supporting Information S1: Section S2.

The second group consists of low‐frequency modes with relatively large imaginary parts of the eigenfrequency. These modes, referred to here as *shell modes*, are primarily localized outside the cavity (see Supporting Information S1: Figure S1) and do not exhibit resonant behavior in the scattering spectra due to their very low Q‐factors. Physically, TE‐ and TM‐polarized shell modes have the same origin as the modes of a sphere made of a perfect electric or magnetic conductor, depending on the polarization [34]. In contrast to the core modes, the shell modes form only a finite series for each polarization and angular momentum ℓ. The spectra of TM‐polarized shell modes with odd ℓ and TE‐polarized shell modes with even ℓ additionally contain a mode with zero real frequency, which we refer to as the *zero‐frequency* mode (see Figure 2). Although the shell modes do not produce characteristic resonant features in the spectra, they play an essential formal role. In particular, they must be included in pole expansions of the electromagnetic Green's function to ensure the completeness of the eigenmode set. For more details, see Supporting Information S1: Section S3.

The coexistence of well‐confined core modes and strongly radiative, delocalized shell modes is a characteristic feature of Mie voids. The mode classification provided above is conditional and not rigorous, especially for low dielectric contrast. Nevertheless, it provides a clear physical picture and correctly explains the evolution of the complex‐valued solutions of Equations (1) and (2) with changes in $n_{\text{bg}}$ and $n_{v}$.

## 3 Results

### 3.1 Spectral Properties of Empty Mie Voids

Before considering Mie voids loaded with excitonic materials, we first gain deeper insight into the properties of a conventional empty Mie void ($\varepsilon_{v}=1$) embedded in a dielectric environment with refractive index $n_{\text{bg}}=\sqrt{\varepsilon_{\text{bg}}}$ (see Figure 1a).

Figure 3 shows the resonant frequencies (the real parts of the complex‐valued eigenfrequencies) as functions of the inverse radius 1/R for several background permittivities (different marker colors), polarizations (TM and TE), angular momenta (ℓ=1,2), and radial mode numbers N. The plot highlights the distinct behavior of resonant frequencies of core and shell modes. Indeed, the spectral position of the core modes (N=1,2) almost does not depend on the background permittivity $\varepsilon_{\text{bg}}$ as previously discussed in ref. [36], whereas the frequency of the shell modes scale as $1/n_{\text{bg}}$ for high values of $n_{\text{bg}}$ (see the Supporting Information S1: Section S3).

**FIGURE 3:**
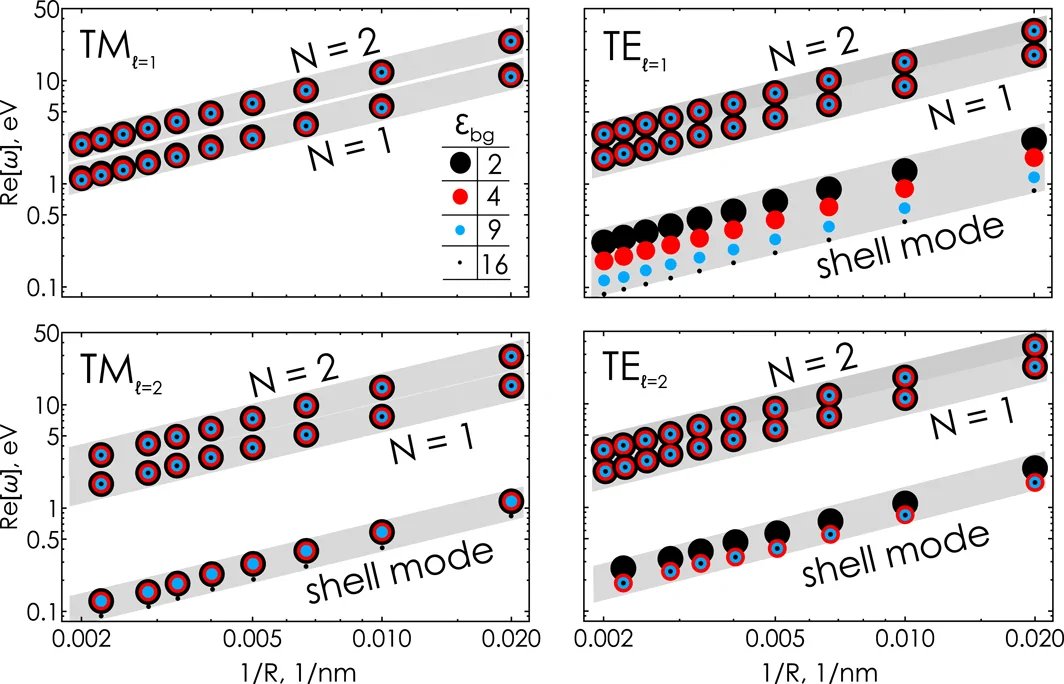
Resonant frequencies of the ${\mathrm{TM}}_{\ell =1,2}$ and ${\mathrm{TE}}_{\ell =1,2}$ quasinormal modes of an empty spherical Mie void surrounded by different transparent dielectrics as functions of the void inverse radius in double logarithmic scale. Gray areas are guides for the eye, approximately indicating the spectral ranges of each particular mode. For the electric field profiles of the presented modes, see Supporting Information S1: Figure S1.

Therefore, the wavelength for such resonances can be substantially larger than the size of the void. However, the use of Mie voids as efficient subwavelength resonators, similar to plasmonic particles, is dubious due to the low Q‐factors of the shell modes. For completeness and illustrative purposes, the imaginary parts of the complex eigenfrequencies of empty Mie voids embedded in dielectrics with $\varepsilon_{\text{bg}}=9,16$ are presented in Supporting Information S1: Figure S2.

The calculated eigenfrequencies ω of empty spherical voids allow us to calculate the quality factors $Q_{v}=Re\left[\omega \right]/2|Im\left[\omega \right]|$. Figure 4 shows the resulting Q‐factors for the dipole and quadrupole void resonances as functions of the background permittivity. Clearly, the Q‐factors grow monotonically with increasing background permittivity, in agreement with findings in [36]. The analytical expressions for spectral positions of Mie‐void resonances and their Q‐factors can be found from Equations (1) and (2) in the limit of high background permittivity as a perturbation series, where $\varepsilon_{v}/\varepsilon_{\text{bg}}\ll 1$ plays a role of small parameter (see the Supporting Information S1: Section S2 for details).

(3) $$\begin{array}{c} TE:\;Q_{v}\approx \frac{z_{0}}{2}\sqrt{\frac{\varepsilon_{\text{bg}}}{\varepsilon_{v}}},\;\;j_{\ell }\left(z_{0}\right)=0, \end{array}$$

(4) $$\begin{array}{c} TM:\;Q_{v}\approx \frac{z_{0}}{2}\left|1-\frac{\ell \left(\ell +1\right)}{z_{0}^{2}}\right|\sqrt{\frac{\varepsilon_{\text{bg}}}{\varepsilon_{v}}},\\ \;z_{0}j_{\ell }^{\prime }\left(z_{0}\right)+j_{\ell }\left(z_{0}\right)=0. \end{array}$$

**FIGURE 4:**
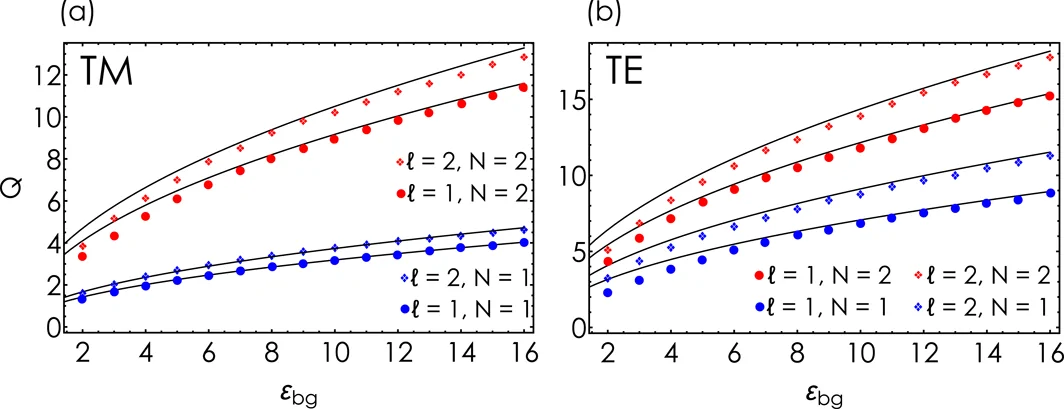
Q‐factors of (a) ${\mathrm{TM}}_{\ell =1,2}$ and (b) ${\mathrm{TE}}_{\ell =1,2}$ modes of a Mie void as functions of the dielectric permittivity of the background material. The black lines represent the analytical expressions for the obtained Q‐factors, Equations (3) and (4).

One can notice that the equations determining the real part of resonant frequencies of TM‐ and TE‐polarized modes match exactly the respective characteristic equations of a spherical PEC cavity [52]. This is expected, since in the limit $\varepsilon_{bg}\to \infty$, the properties of the dielectric background medium approach those of the perfect electric conductor. The resulting analytical expressions agree well with the numerical results for both TM and TE modes, even for moderately small background permittivities ($\varepsilon_{\text{bg}}≳2$) (see Figure 4). It is interesting to note the extremely slow growth of Q‐factors of the core modes with $\varepsilon_{bg}$ in contrast to the much faster growth of Q‐factors of a spherical dielectric particle in air [53].

Recently, a detailed theoretical analysis of Mie voids was reported in ref. [49]. In that work, the authors employed approximate analytical representations of the Riccati–Bessel functions to obtain approximate asymptotics expressions for the complex‐valued eigenfrequencies. In contrast, our approach constructs the asymptotic series directly from the exact roots of the Riccati–Bessel functions, which leads to a more compact formulation involving only a single small parameter, namely the dielectric contrast between the cavity and the environment, $n_{v}/n_{\text{bg}}\ll 1$. In addition, our analytical treatment provides explicit expressions not only for the core modes (Mie resonances) but also for the eigenfrequencies of the shell modes.

### 3.2 Mie Void Loaded With a Resonant Medium

Next we analyze the polaritonic system (see Figure 1b), by filling the spherical void with a resonant medium described by the single‐pole Lorentz model.

(5) $$\varepsilon \left(\omega \right)=\varepsilon_{\infty }+f\frac{\omega_{0}^{2}}{\omega_{0}^{2}-\omega^{2}-i\gamma_{\text{ex}}\omega },$$

where $\varepsilon_{\infty }$ is the high‐frequency permittivity, f is the reduced oscillator strength, $\omega_{0}$ and $\gamma_{\text{ex}}$ are the polaritonic resonance frequency and decay rate.

Substituting Equation (5) into Equations (1) and (2) with $\varepsilon_{v}=\varepsilon \left(\omega \right)$, and linearizing the resulting expressions in the vicinity of $\omega \approx \omega_{v}\approx \omega_{0}$, we obtain the following compact equation for the polaritonic spectrum:

(6) $$\left(\omega -\omega_{v}+i\gamma_{v}/2\right)\;\left(\omega -\omega_{0}+i\gamma_{\text{ex}}/2\right)=\frac{f\;\omega_{0}^{2}}{4\varepsilon_{\infty }}.$$

Here, the first bracket corresponds to the geometrically scalable Mie‐void resonance, where $\omega_{v}$ and $\gamma_{v}$ are the Mie‐void resonant frequency and the decay rate, respectively, different for TM‐ and TE‐polarized modes (see Supporting Information S1: Section S4 for details). The second bracket describes the bulk excitonic resonance, and the right‐hand side encodes coupling between exciton and Mie resonance with an effective coupling constant.

(7) $$g=\left(\omega_{0}/2\right)\sqrt{f/\varepsilon_{\infty }}.$$

This expression agrees with those from ref. [24], confirming that the bulk coupling strength can be taken as a reasonable estimate of the collective coupling strength for spherical resonators loaded with a resonant medium. Equation (6) is equivalent to the eigenvalue problem of an effective 2×2 non‐Hermitian Hamiltonian,

(8) $$\hat{H}=\left(\begin{array}{cc} \omega_{0}-i\gamma_{\text{ex}}/2 & g\\ g & \omega_{v}-i\gamma_{v}/2 \end{array}\right).$$

The eigenfrequencies of this effective Hamiltonian are given by the following equation:

(9) $$\begin{array}{c} \omega_{\pm }=\frac{\omega_{v}+\omega_{0}}{2}-\frac{i}{2}\left(\frac{\gamma_{\text{ex}}}{2}+\frac{\gamma_{v}}{2}\right)\\ \pm \sqrt{g^{2}+\frac{1}{4}{\left(\omega_{0}-\omega_{v}-i\left(\frac{\gamma_{\text{ex}}}{2}-\frac{\gamma_{v}}{2}\right)\right)}^{2}}. \end{array}$$

The Rabi splitting $\Omega =\omega_{+}-\omega_{-}$ between the two polaritonic states at zero detuning ($\omega_{v}=\omega_{0}$) is real‐valued if $4g>\left|\gamma_{\text{ex}}-\gamma_{v}\right|$.

Figure 5a,b show numerically and analytically calculated polaritonic spectra of TM‐ and TE‐polarized dipolar modes of the void filled with a resonant medium (f=0.1, $Q_{\text{ex}}=100$) and surrounded by a transparent dielectric ($\varepsilon_{bg}=16$). For the sake of simplicity, we set $\varepsilon_{\infty }=1$. The analytical spectra (see Equation (6)) shown by solid curves are in good agreement with the numerical results (markers) both on and off excitonic resonance. The spectra clearly exhibit anti‐crossings, indicating strong coupling between the void mode and the excitonic transition. With increasing radial number N, the anticrossing shifts to larger void radii (smaller $\lambda_{0}/R$), consistent with electromagnetic frequency scaling. Figure 5c shows the corresponding spectra for a weaker oscillator strength (f=0.01, $Q_{\text{ex}}=100$), where both weak and strong coupling regimes appear depending on the optical mode. Each photonic mode generates an independent pair of lower and upper polaritons [54, 55], reflecting the spatially nonuniform exciton–photon coupling. This contrasts with systems featuring localized point‐like transitions, where multiple photonic modes couple to a single resonance and produce middle polaritons [56].

**FIGURE 5:**
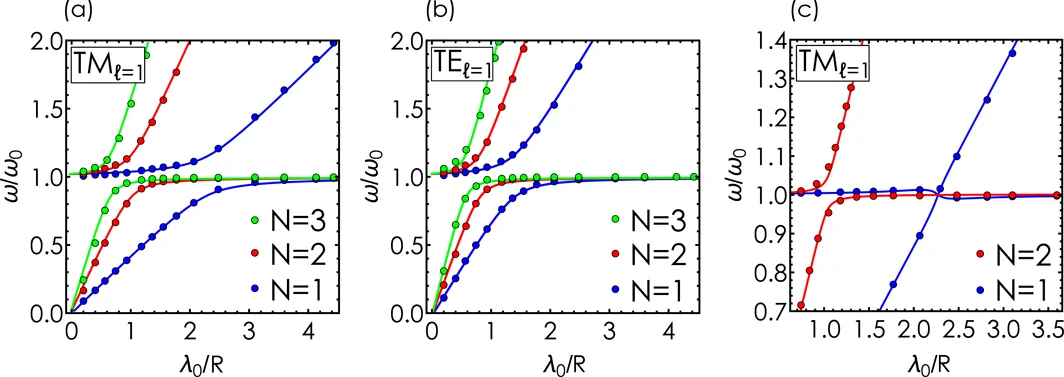
Polaritonic spectra of a Mie void surrounded by a transparent dielectric ($\varepsilon_{bg}=16$) and loaded with a resonant medium. (a), (b) Eigenfrequency spectra of the ${\mathrm{TM}}_{\ell =1}$ and ${\mathrm{TE}}_{\ell =1}$ modes of the dielectric void loaded with a resonant medium (f=0.1, $Q_{\text{ex}}=100$), revealing a set of anti‐crossings. (c) Same as (a) but for f=0.01, $Q_{\text{ex}}=100$. $\lambda_{0}=2\pi c/\omega_{0}$ is the resonant wavelength of the Lorentz material. The colored curves represent the analytical polaritonic spectra, Equation (6).

Expressing the coupling constant as Equation (7) in terms of the Lorentz oscillator model, we arrive at the following criterion of observing real‐valued Rabi splitting between the two eigenstates of the coupled system (polariton anti‐crossing):

(10) $$f>{\left(\frac{1}{2Q_{\text{ex}}}-\frac{1}{2Q_{v}}\right)}^{2},$$

where $Q_{v}$ is the quality factor of the void cavity, which can be expressed through $\varepsilon_{bg}$ by analytical expressions in Equations (3) and (4).

Equation (10) allows us to introduce the *threshold* value of f delimiting the regions of weak and strong coupling in terms of polaritonic frequency splitting for the given parameters:

(11) $$f_{\text{th}1}={\left(\frac{1}{2Q_{\text{ex}}}-\frac{1}{2Q_{v}}\right)}^{2}.$$

Figure 6a shows the regions of weak (white) and strong (colored) coupling defined according to Equation (10) for the TM‐polarized dipolar N=2 mode in the parameter space of the background permittivity and the reduced oscillator strength f. Analytical approximation, Equations (3) and (4), was used to evaluate $Q_{v}$. Higher values of background permittivity favor the emergence of anticrossing in this system until the point of equal Q‐factors, $Q_{v}=Q_{\text{ex}}$ (black‐dashed circle in Figure 6a), where polaritonic frequency anticrossing takes place for arbitrarily low values of f, Equation (10). Past this point of equal Q‐factors increasing the background refractive index (and the void mode Q‐factor, respectively) requires higher oscillator strength to achieve frequency splitting. This trend may seem counter‐intuitive; however, it is completely in agreement with strong coupling physics [57]—achieving splitting of complex eigenfrequencies at zero detuning is easiest with equal Q‐factors of the two oscillators, as one can see directly from Equation (10).

**FIGURE 6:**
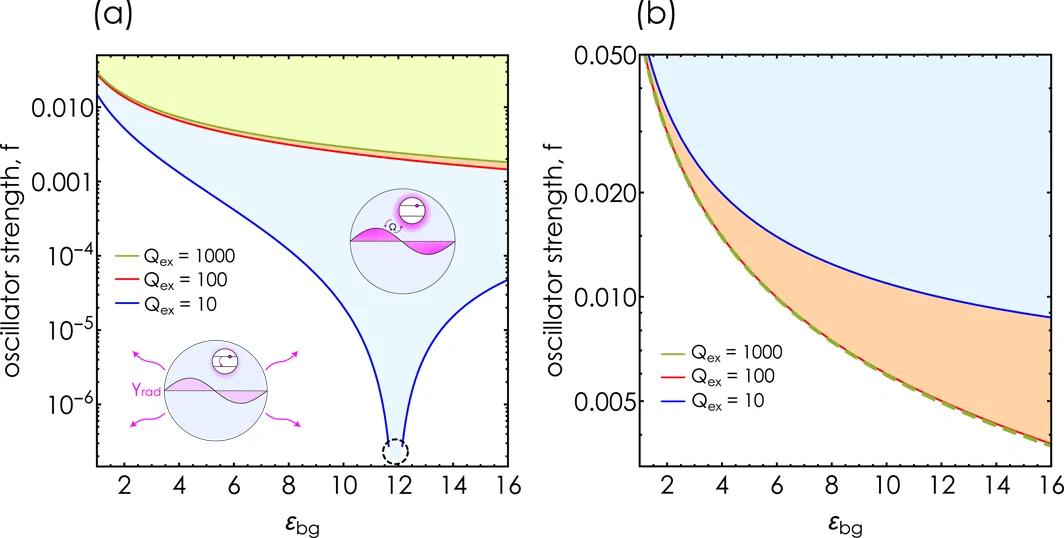
(a) Coupling diagram in the parameter space of the background dielectric permittivity $\varepsilon_{bg}$ and reduced oscillator strength f in logarithmic scale for various Q‐factors of the resonant medium filling the void. The lines indicate the corresponding threshold values of reduced oscillator strength $f_{\text{th}1}$; the colored areas depict strong coupling domains between the ${\mathrm{TM}}_{\ell =1}\;N=2$ cavity mode and the resonant medium in accordance with Equation (10). Insets are a graphical illustration of weak and strong coupling in Mie voids loaded with resonant media. Black dashed circle marks the point of Q‐factors matching, $Q_{\text{ex}}=Q_{v}$, resulting in strong coupling for arbitrarily low values of f. (b) Same as (a) but for the stronger threshold $f_{\text{th}2}$, Equation (13).

We note that criterion Equation (10) only guarantees polaritonic splitting of complex eigenfrequencies in the complex frequency plane [57]. This splitting, however, is not easy to observe spectroscopically. On the other hand, in order to observe an anticrossing in a spectral response of the system (such as extinction cross‐section), the system has to follow a more stringent condition of Rabi splitting Ω exceeding the polariton half‐width at half maximum [11, 58].

(12) $$\Omega >\frac{\gamma_{\text{ex}}}{2}+\frac{\gamma_{v}}{2}.$$

This inequality leads to a stronger criterion for observation of polariton anticrossing.

(13) $$f>f_{\text{th}2},\;f_{\text{th}2}=\left(\frac{1}{2Q_{\text{ex}}^{2}}+\frac{1}{2Q_{v}^{2}}\right).$$

The respective coupling diagrams for three different quality factors $Q_{\text{ex}}$ of the resonant medium are presented in Figure 6b. For this system, higher values of $\varepsilon_{bg}$ guarantee lower values of $f_{\text{th}2}$. The two criteria tend to show identical coupling regions in the limit of high $Q_{\text{ex}}$ and $Q_{v}$: vanishingly small oscillator strength places the system in the strong coupling regime.

In addition, Figure 7 shows the evolution of partial and total scattering cross‐sections of a Mie void loaded with a Lorentz medium in different coupling regimes. For f below the strong coupling threshold, Figure 7a, cross‐sections do not show any signs of light–matter coupling, as expected. Exactly at the strong coupling threshold, $f=f_{\text{th}2}$, all partial and total cross‐sections start to show signs of coherent light–matter coupling, as evidenced by the development of a Fano resonance around the exciton frequency $\omega_{0}$, Figure 7b [59, 60, 61, 62]. Finally, deep in the strong coupling regime, $f\gg f_{\text{th}2}$, the Fano resonance deepens in all partial cross‐sections; nonetheless, clear mode splitting is hard to identify even in this regime. This is typical for multimode structures: even partial cross‐section for a fixed polarization and angular momentum accounts for multiple Fabry–Perot‐like Mie modes, whose presence often does not allow observing a clear anti‐crossing in extinction or scattering spectra [27].

**FIGURE 7:**
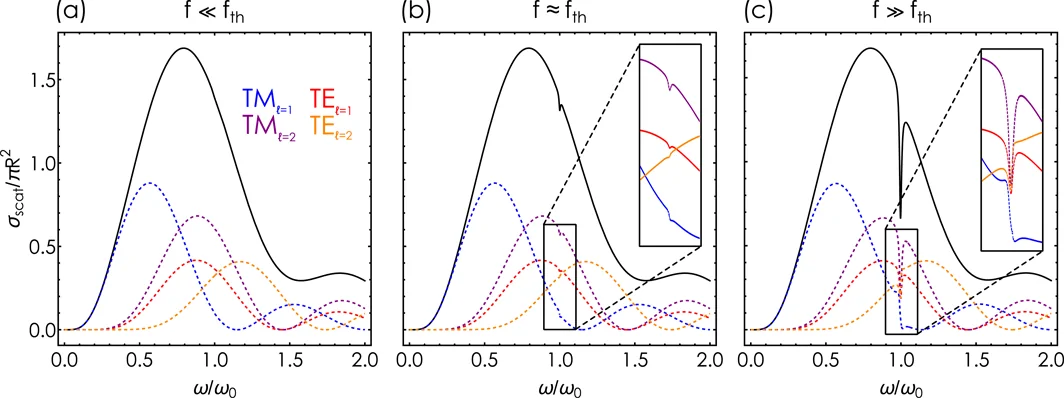
The effect of the Lorentz oscillator strength on the scattering cross‐section of a Mie void. (a) Partial and total cross‐section of a Mie void for $\varepsilon_{bg}=16$ loaded with a resonant medium with $Q_{\text{ex}}=100$ and $f=5\cdot 10^{-4}$ below the strong coupling threshold ($f_{\text{th}2}$, Equation (13) of the ${\mathrm{TM}}_{\ell =1,2}\;N=1$ and ${\mathrm{TE}}_{\ell =1,2}\;N=1$ modes. (b) Same as (a) for $f=5\cdot 10^{-3}$ slightly above the strong coupling threshold of the ${\mathrm{TE}}_{\ell =2}\;N=1$ mode. (c) Same as (a) for f=0.1 way above the strong coupling threshold of all the presented modes.

### 3.3 Empty Mie Void Surrounded by a Dispersive Medium

Finally, we examine the eigenfrequency spectra of an empty spherical void ($\varepsilon_{v}=1$) embedded in a dispersive resonant medium, Figure 1c, described by the Lorentz model, Equation (5). To obtain a representative range of oscillator strength values of the high‐refractive index background medium, we fit the complex‐valued permittivity of crystalline silicon from ref. [63] with a set of Lorentz poles:

(14) $$\varepsilon \left(\omega \right)=\varepsilon_{\infty }+\sum_{n}f_{n}\frac{\omega_{n}^{2}}{\omega_{n}^{2}-\omega^{2}-i\gamma_{n}\omega }.$$

The resulting fit is shown in Supporting Information S1: Figure S3 with the corresponding reduced oscillator strength values. According to these values, we extend the range of the oscillator strength beyond f=1 in the single‐pole model (Equation (5)), and set $\varepsilon_{\infty }=16$ for the following calculations.

Figure 8 shows the behavior of complex‐valued eigenfrequencies of an empty spherical void in a resonant background for the cases of low‐Q, $Q_{\text{ex}}\approx Q_{v}$ (top row), and high‐Q, $Q_{\text{ex}}\gg Q_{v}$ (bottom row) resonant media. The results were obtained for the first three radial numbers N=1,2,3 of a TM‐polarized ℓ=1 mode.

**FIGURE 8:**
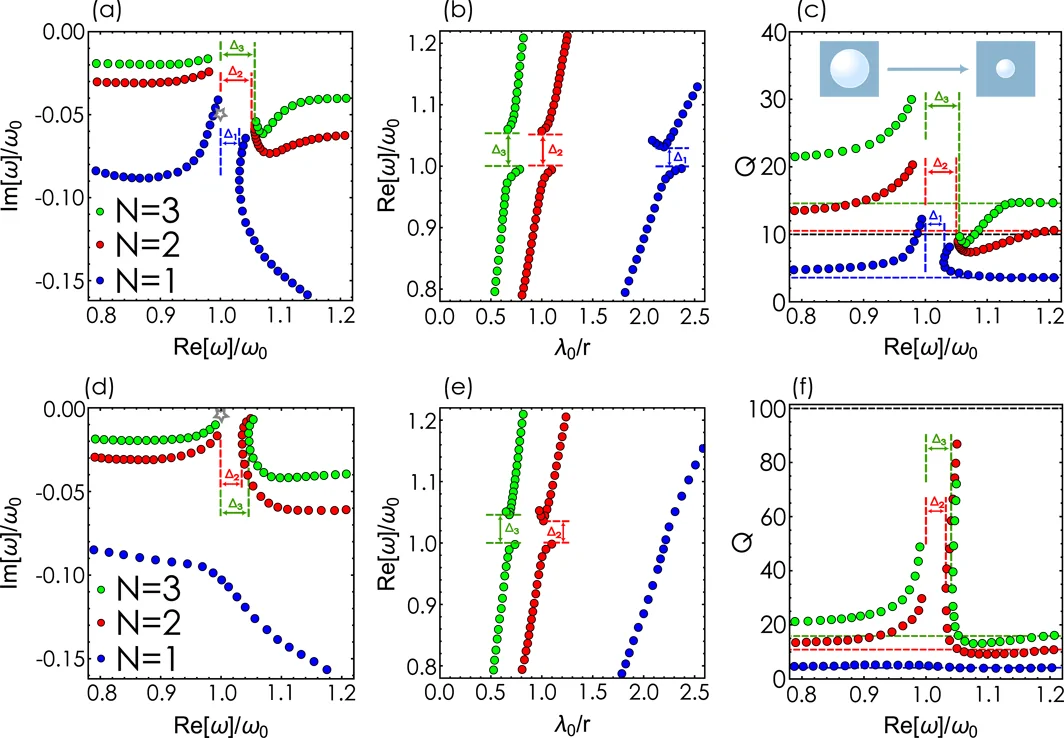
The effect of the background resonant medium on the behavior of eigenfrequencies and Q‐factors of a Mie void. The results were obtained for the ${\mathrm{TM}}_{\ell =1}$ mode, low‐Q (top row, $Q_{\text{ex}}=10$) and high‐Q (bottom row, $Q_{\text{ex}}=100$) resonant background media with f=2 and $\varepsilon_{\infty }=16$. (a) Trajectories of polaritonic eigenfrequencies in the complex frequency plane for the N=1,2,3 modes; the gray star indicates the background medium resonant frequency. $\Delta_{i}$ marks the position of a respective polariton gap for each mode. (b) Real parts of eigenfrequencies from (a) plotted as a function of the normalized inverse radius of the Mie void. (c) Q‐factors of polaritonic modes as functions of the normalized resonant frequency. The horizontal black and colored dashed lines mark the quality factors of the background medium and photonic modes, respectively. (d)–(f) Same as (a)–(c) but evaluated for the case of a high‐Q ($Q_{\text{ex}}=100$) background medium. Inset in (c) shows the direction of the void radius sweep.

It is instructive to analyze the eigenfrequency trajectories in the complex frequency plane, Figure 8a,d. For some sets of parameters, the eigenfrequencies demonstrate splitting into upper (UP) and lower (LP) polariton branches in the vicinity of the excitonic complex frequency $\omega_{0}-i\gamma_{\text{ex}}/2$ (gray stars), except for the TM‐polarized ℓ=1, N=1 mode, Figure 8d. Particularly low Q‐factor of the N=1 TM‐polarized dipole core mode is not sufficient to induce complex frequency mode splitting. Interestingly, a recent study [49] also reported frequency anticrossings in a dielectric void. Specifically, they have demonstrated two anticrossing mechanisms: the first is that related to the through‐the‐continuum (far‐field) interaction of two nonorthogonal purely photonic resonances [64, 65]. In addition, the authors also did briefly discuss hybridization of a purely photonic mode with a material pole (see Figure 5 in ref. [49]) and the formation of two disconnected polaritonic branches.

The pairs of LP and UP that exhibit anticrossing additionally feature the formation of polariton gaps, Δ [66]. The gap widens with increasing radial number N. These gaps in polaritonic structures always originate on the blue side of the material resonances, and their origin can be understood from the appearance of the reststrahlen band in the permittivity of the resonant material [66, 67]. The next column, Figure 8b,e, for convenience displays the same set of data plotted in the coordinates of normalized resonant frequency $Re\left[\omega \right]/\omega_{0}$ and normalized inverse radius $\lambda_{0}/R$, where $\lambda_{0}=2\pi c/\omega_{0}$ is the resonant wavelength of the Lorentz material.

Polariton gap appears in the spectrum of a hybrid system when the photonic frequency varies continuously across the resonant material frequency. In systems possessing translational invariance, the upper edge of the polariton gap can be calculated as the energy of the upper polariton in the limit of k→0, $\bar{\omega }=\sqrt{\omega_{0}^{2}+4g^{2}}$ [66]. Conversely, the lower edge can be calculated as the energy of the lower polariton in the limit of k→∞, which is exactly the uncoupled excitonic resonant frequency $\omega_{0}$ [66]. In order to vary bare photonic frequency in a compact polaritonic structures—such as the one studied here—in a the continuous range, the in‐plane wave‐vector component can be replaced with the inverse characteristic dimension of the system, $k_{\|}\to 1/R$. Figure 8b,e show that the lower edge of the polariton gap indeed coincides with the frequency of the uncoupled resonant oscillations of the background medium. However, the upper edge of the gap does not occur at r→∞, and instead occurs at intermediate values of r. Furthermore, the spectra of the upper polariton display a characteristic back‐bending picture [see N=1 mode in Figure 8b and N=2,3 modes in Figure 8e].

Next, we analyze this data from the perspective of the Q‐factors of the resulting modes, Figure 8c,f, which contains the most intriguing results. The observed splitting is accompanied by an increase in the quality factor of the resulting polaritonic modes in comparison with the uncoupled photonic void modes. We note that the behavior of polaritonic Q‐factors is qualitatively different depending on the ratio between $Q_{v}$ and $Q_{\text{ex}}$. In the case of low‐Q resonant medium, $\gamma_{\text{ex}}\approx \gamma_{v}$, the Q‐factor of the lower polariton mode exceeds both the photonic *and* the excitonic Q‐factors, Figure 8c. This behavior is similar to the observation of polariton linewidth asymmetry in structures incorporating plasmonic meta‐atoms [68]. In the case of high‐Q resonant medium, $\gamma_{v}\gg \gamma_{\text{ex}}$, the Q‐factors of both polariton modes exceed the bare cavity Q‐factor only and approach that of the exciton, Figure 8f.

This kind of Q‐factor improvement has also been discussed by Hentschel et al. [36]. The authors explained this effect by the greater refractive index mismatch in the region of absorption lines of the background medium. Our polaritonic picture of light–matter interaction in this class of systems offers an alternative partial interpretation of this behavior.

To illustrate the polaritonic nature of the quality factor improvement, we analyze the Q‐factor dynamics from the perspective of the coupled oscillators Hamiltonian, Equation (8). To that end, we show in Fig. S4 the Q‐factors obtained from Equation (9) for the same set of cavity mode and Lorentz resonance parameters; as a reasonable estimation, the value of the coupling strength was determined by Equation (7). The results of the 2×2 effective Hamiltonian model do qualitatively agree with the exact Mie solution in the case of high‐Q resonant background, $\gamma_{\text{ex}}\ll \gamma_{v}$ (see Supporting Information S1: Figure S4d,e,f). Within the 2×2 model, the two polaritons equalize their decay rates at the zero detuning: $\gamma_{UP}=\gamma_{LP}=\left(\gamma_{v}+\gamma_{\text{ex}}\right)/2$ (see Equation (9)). Under the condition $\gamma_{\text{ex}}\ll \gamma_{\mathrm{cav}}$, this causes both polaritonic modes to acquire lower decay rates compared to the uncoupled cavity mode, $\gamma_{LP,UP}\approx \gamma_{\text{ex}}/2$. However, close to the polariton gap the Q‐factors increase even further beyond this simple factor‐2 improvement. Indeed, close to the gap, the upper and lower polariton modes become purely excitonic, and acquire the corresponding Q‐factor, $Q_{UP}\approx Q_{LP}\approx Q_{\text{ex}}$.

On the other hand, there is almost no correspondence between the exact Mie solution and the 2×2 model in the case of low‐Q resonant medium, $\gamma_{\text{ex}}\approx \gamma_{v}$ (see Supporting Information S1: Figure S4a,b,c). We attribute this to the strongly inhomogeneous field distribution of the quasinormal modes of the spherical voids. Indeed, the value of the normalized vacuum electric field E of the void QNM becomes position‐dependent within the resonant medium, E=E(r). This leads to position‐dependent coupling strength g∝E⋅μ with μ being the elementary transition dipole moment of the Lorentz oscillator, which invalidates the simple 2×2 model. Another probable explanation of this behavior lies in the real‐valued nature of the coupling constant assumed in the 2×2 model (8). The radiative line narrowing is known to occur in resonant systems, where a few optical modes interfere destructively through a single scattering channel. This is commonly described by the imaginary part of the coupling constant [69, 70, 71]. This through‐the‐continuum interaction typically leads to the formation of a super‐ [72] and sub‐radiant modes, commonly referred to as quasi‐BICs [64, 65, 70, 73, 74]. The imaginary part of the coupling constant can be introduced phenomenologically or through a more accurate expansion of Equations (1) and (2) up to higher‐order terms. The breakdown of the Taylor series expansion used to obtain the eigenfrequencies of an empty core mode could also be one of the reasons.

Similarly to the analysis in Section 2.2, we plot the regions of weak and strong coupling in terms of anti‐crossing in the complex frequency plane (see Equation (10)) in the $\varepsilon_{\infty }$‐f parameter space for the TM‐polarized ℓ=1, N=2 mode and 2 different quality factors of the background medium, $Q_{\text{ex}}=10$ and $Q_{\text{ex}}=100$, Figure 9.

**FIGURE 9:**
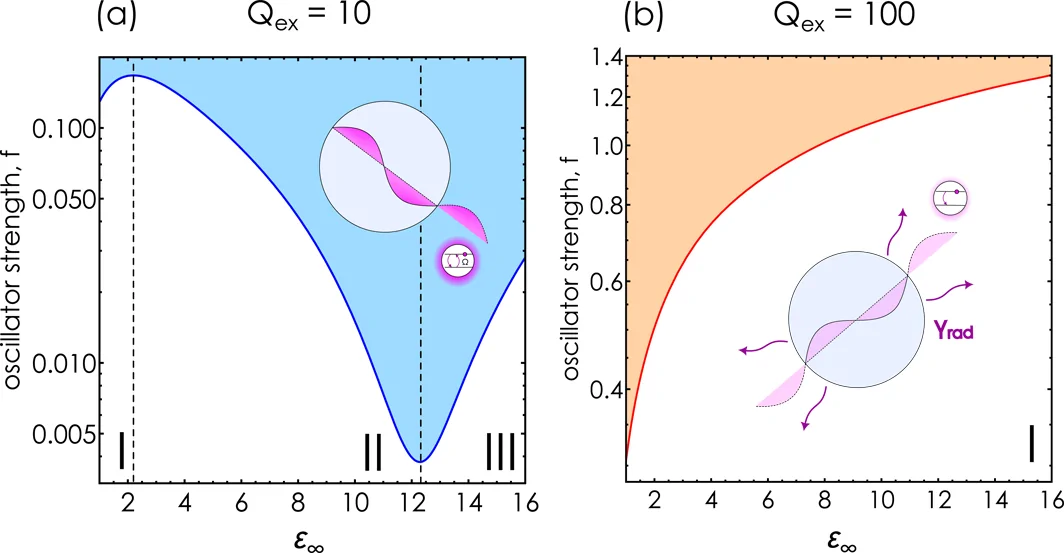
Coupling diagrams in the parameter space of the high‐frequency background permittivity $\varepsilon_{\infty }$ and reduced oscillator strength f in logarithmic scale. The colored and white areas show the numerically resolved strong and weak coupling regimes between the ${\mathrm{TM}}_{\ell =1}\;N=2$ cavity mode and the (a) low‐Q and (b) high‐Q resonant background media. Insets: free interpretation of weak and strong coupling in Mie voids embedded in resonant medium.

The resulting diagrams display a more complicated behavior compared to those for a cavity loaded with resonant media in a transparent background. The transition between regions II and III (where the strong coupling region meets the f = 0 axis) is analogous to that in medium‐loaded cavities see Figure 6a: increasing $\varepsilon_{\infty }$ improves the photonic mode Q‐factor and favors the emergence of anticrossing, thus reducing threshold value of f, until the system reaches the point of equal Q‐factors, where $f_{\text{th}}$ approaches 0, Figure 9a.

However, in contrast to the results presented in Figure 6a, here we observe an unexpected transition to region I at low $\varepsilon_{\infty }$, where $f_{\text{th}}$ again grows with $\varepsilon_{\infty }$. In contrast to the empty Mie void surrounded by a transparent background, the resonant background medium here interacts with the exponentially diverging tails of the quasinormal mode. On one hand, low $\varepsilon_{\infty }$ deteriorates the photonic Q‐factors and should intuitively lead to increasing $f_{\text{th}}$. However, the fields of the mode itself become even more delocalized and penetrate further into the background where the interaction with the resonant medium occurs. This argument could explain the observed behavior of the threshold value of oscillator strength in region I. In the case of the resonant medium with a higher Q‐factor, Figure 9b, we observe a part of region I accompanied by growing $f_{th}$ with $\varepsilon_{\infty }$. Transition to regions II and III in this case also does occur, but at much greater $\varepsilon_{\infty }$ yielding appropriate photonic Qs.

The presence of frequency dispersion in the background affects not only the spectrum of polaritonic frequencies but also the spatial behavior of the resulting QNMs. Typical problems involving dielectric spherical particles imply a transparent background medium, resulting in exponentially diverging fields in space [45]. The radial part of the QNM field supported by the spherical cavity is described by the spherical Hankel functions of the first kind, $h_{\ell }^{\left(1\right)}\left(kr\right)$, which in the far‐field zone asymptotically behave as follows:

(15) $$h_{\ell }^{\left(1\right)}\left(kr\right)\to {\left(-i\right)}^{\ell +1}\frac{e^{\mathrm{ikr}}}{kr}.$$

Absorption in the background medium may result in spatial localization of the QNM fields. Specifically, localization occurs when Im[k]>0:

(16) $$\begin{array}{cc} & Im\left[\left(\omega^{\prime }+i\omega^{\prime \prime }\right)\right.\\ & \left.\sqrt{\varepsilon_{\infty }+\frac{f\omega_{0}^{2}}{\omega_{0}^{2}-{\left(\omega^{\prime }+i\omega^{\prime \prime }\right)}^{2}-i\gamma_{\text{ex}}\left(\omega^{\prime }+i\omega^{\prime \prime }\right)}}\right]>0. \end{array}$$

This expression clearly highlights the competition between two factors determining the possible localization of the QNM: negative imaginary part of the QNM eigenfrequency $\omega^{\prime \prime }<0$ leading to a spatially diverging behavior, and the positive imaginary part of the refractive index of the Lorentz medium $Im\sqrt{\varepsilon }>0$, leading to spatial localization.

Figure 10 shows the regions of QNM localization in the complex frequency plane for a set of the resonant medium oscillator strengths f and the exciton quality factors, defined by Equation (16). Clearly, larger values of the oscillator strength f and the decay rate $\gamma_{\text{ex}}$ of the background medium favor QNM localization. The localization regions do not extend beyond $-\gamma_{\text{ex}}/2$ (gray stars) along the imaginary frequency axis. Each panel of Figure 10 additionally demonstrates the trajectories of polaritonic eigenfrequencies for TM‐polarized ℓ=1, N=2,3 modes overlayed on top of the localization regions. Panels (a, b, c) of Figure 10 show that the solutions on the lower polariton branch in the case of the low‐Q resonant medium regime enter the localization region in the vicinity of resonant frequency in the limit of r→0. This, however, does not occur in the opposite high‐Q resonant medium regime, Figure 10d,e,f. Generally, for such a polaritonic mode to become localized, one needs to combine a high‐Q photonic mode with a low‐Q resonant medium.

**FIGURE 10:**
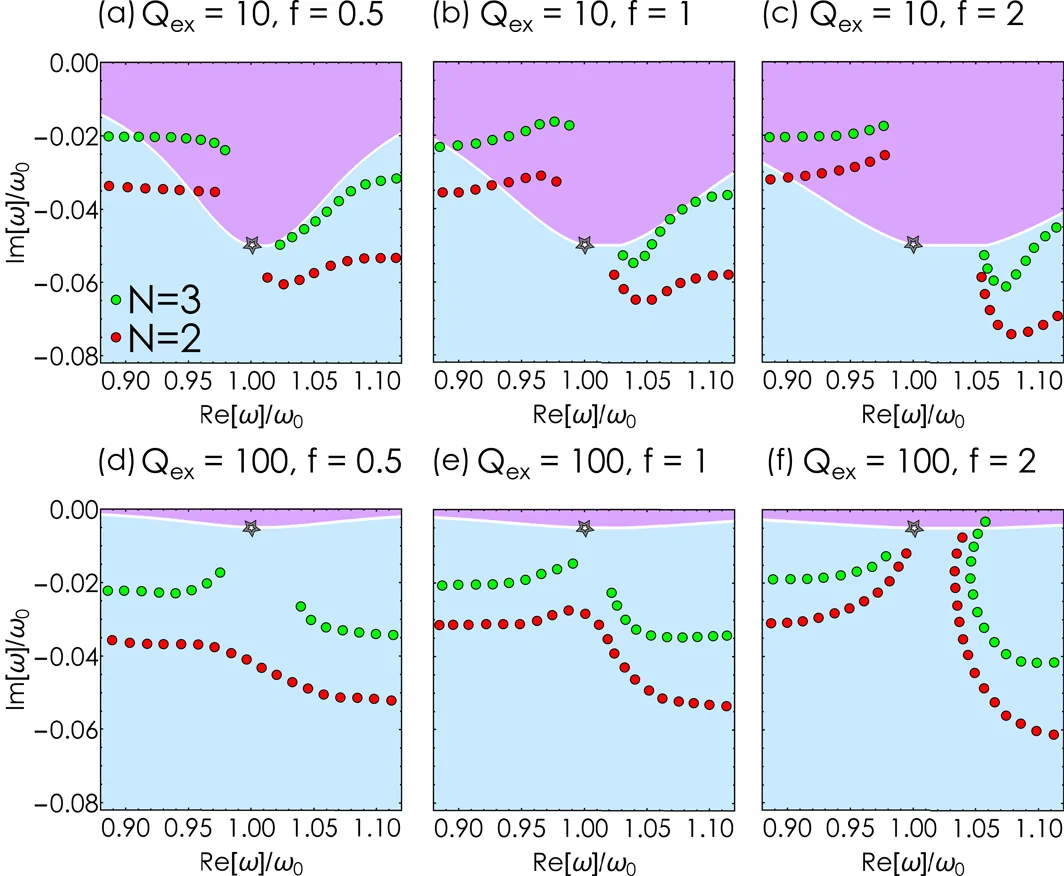
QNM localization diagrams in the normalized complex frequency parametric space for a set of resonant background quality factors and oscillator strengths, $\varepsilon_{\infty }$ = 16. Magenta (cyan) areas indicate where the solutions present localized (delocalized) fields. The gray stars represent the positions of the medium resonances, and the overlayed polaritonic eigenfrequency trajectories represent the ${\mathrm{TM}}_{\ell =1}\;N=2,3$ modes with the thick colored curves to lead the eye.

Finally, we present in Figure 11 spatial electric field cross‐sections for a few selected QNMs of an empty spherical void in a resonant absorbing background demonstrating either delocalized (DL) or localized (L) spatial behavior for the sake of illustration. The presented localized modes correspond to the lower‐polariton solutions resulting from Mie theory, with eigenfrequencies consistent with the condition presented by Equation (16). This clearly shows that polariton anticrossing and Q‐factor enhancement resulting from strong coupling between the cavity modes of a Mie void and the background material are partially responsible for spatial field localization for certain sets of parameters. The exact frequency and medium parameter values used in Figure 11 are presented in Supporting Information S1: Table S1.

**FIGURE 11:**
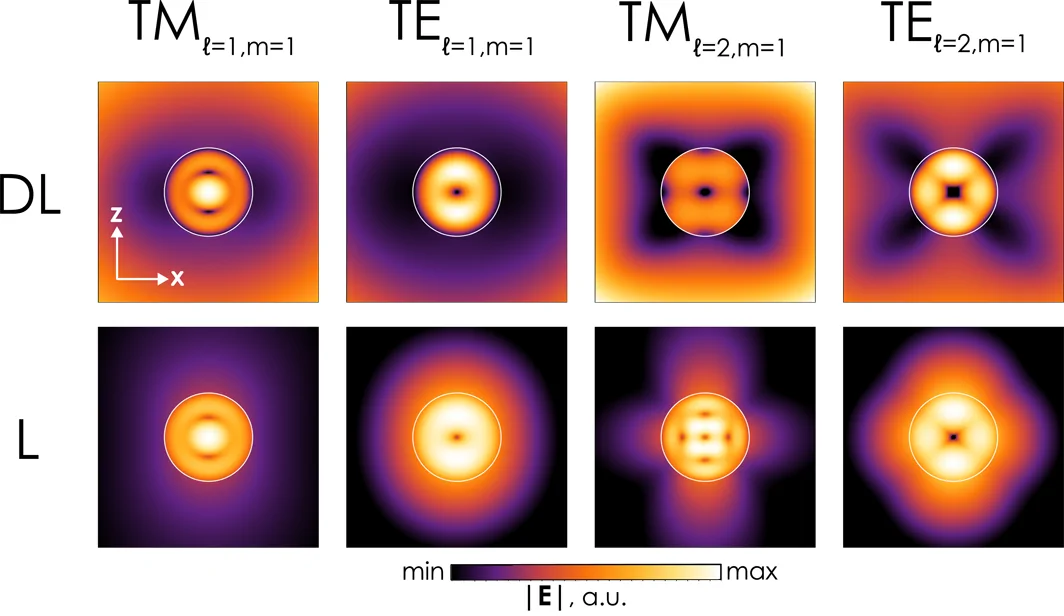
Field profiles for delocalized (DL, top row) and localized (L, bottom row) quasinormal modes of Mie voids surrounded by a dispersive dielectric background, Equation (5). Table S1 of SI contains the numerical values of frequency and medium parameters used for plotting the field distribution of each particular quasinormal mode.

## 4 Conclusion

To conclude, we have investigated the spectral properties of optical Mie voids in three distinct scenarios: (i) empty voids in transparent dielectrics, (ii) voids loaded with resonant media, and (iii) empty voids embedded in dispersive resonant media. The eigenfrequencies of Mie voids in nondispersive media are almost independent of the host refractive index as long as it is higher than that of the voids. The Q‐factors of Mie voids (in fact, of generic high‐refractive‐index‐contrast spherical cavities) obey analytical equations similar to those of spherical PEC cavities.

When the void is filled with a resonant medium, the system exhibits both weak‐ and strong‐coupling regimes depending on the radial order of the mode, as confirmed analytically and by numerical simulations. Generally, a higher refractive index contrast at the interface between the void loaded with resonant media and the transparent host medium makes the system less demanding for reaching strong coupling.

In the case of empty Mie voids embedded in a dispersive resonant medium, the quasinormal modes penetrating the host material demonstrate the polariton anticrossing with the formation of polariton gaps generally increasing with the radial mode number. The interaction between the void eigenmodes and the host media resonances leads to the enhancement of the polariton Q‐factors, even exceeding those of the bare components in the case of matching individual Q’s, probably owing to the through‐the‐continuum nature of their interaction. Moreover, the combination of absorption in the host medium and the Q‐enhanced polariton anticrossing can lead to the QNM localization governed by the complex eigenfrequencies, as well as the material parameters. This effect can be used for spatially localizing the modes that would not otherwise have been localized in certain systems.

Our theoretical framework establishes Mie voids as a versatile platform for polaritonic engineering, offering distinct advantages in high‐permittivity environments. The scaling of Q‐factors with background permittivity provides a natural route to suppress radiative losses, whereas the onset of strong coupling positions Mie voids as a prospective platform for polaritonic applications. Our results highlight the potential of Mie voids in all‐dielectric nanophotonics, where conventional cavity designs face fundamental limitations.

## Author Contributions

D.G.B. conceived the idea of the study. E.R. and. A.A.B. performed theoretical analysis. E.R. and D.G.B. wrote the manuscript with contribution from all authors.

## Funding

The work was supported by the Ministry of Science and Higher Education of the Russian Federation (Grant FSMG‐2024‐0014). Theoretical analysis of Q‐factors of polaritonic voids was partially supported by Russian Science Foundation (Grant 23‐72‐10005). A.A.B. acknowledges support from the National Natural Science Foundation of China (Project W2532010) and the academic leadership program Priority 2030.

## Ethics Statement

The authors have nothing to report.

## Conflicts of Interest

The authors declare no conflicts of interest.

## Supporting information


Supporting Information S1

## Data Availability

The datasets generated and/or analyzed during the current study are available from the corresponding author upon reasonable request.
