# Supplementary material for: Polaritonic Spectra of Optical Mie Voids
Source: Nanophotonics. 2026 Apr 23;15(9):e70097. doi: 10.1002/nap2.70097 (PMC13642536; doi:10.1002/nap2.70097)
Supplement: Supplementary file 1 [file NAP2-15-e70097-s001.pdf]

Supporting Information for "Polaritonic spectra of optical Mie voids"

Evgeny Ryabkov,[1,2] Mingzhao Song,[1,*] Andrey A. Bogdanov,[1,3,†] and Denis G. Baranov[2,‡]

[1] *Qingdao Innovation and Development Center of Harbin Engineering University, Qingdao 266000, Shandong, China*

[2] *Center for Photonics and 2D Materials, Moscow Institute of Physics and Technology, Dolgoprudny 141700, Russia*

[3] *ITMO University, St. Petersburg 197101, Russia*

CONTENTS

S1. Additional data 2

S2. Resonances and Q-factor of Mie Voids in a high-refractive-index media 5

A. TM modes 5

B. TE modes 7

C. Analytical expressions for Q-factors 7

S3. Low-Q modes of Mie voids 8

S4. POLARITONIC SPECTRA OF MIE VOIDS FILLED WITH RESONANT MEDIUM 9

* kevinsmz@foxmail.com

† a.bogdanov@hrbeu.edu.cn

‡ denis.baranov@phystech.edu

S1. ADDITIONAL DATA

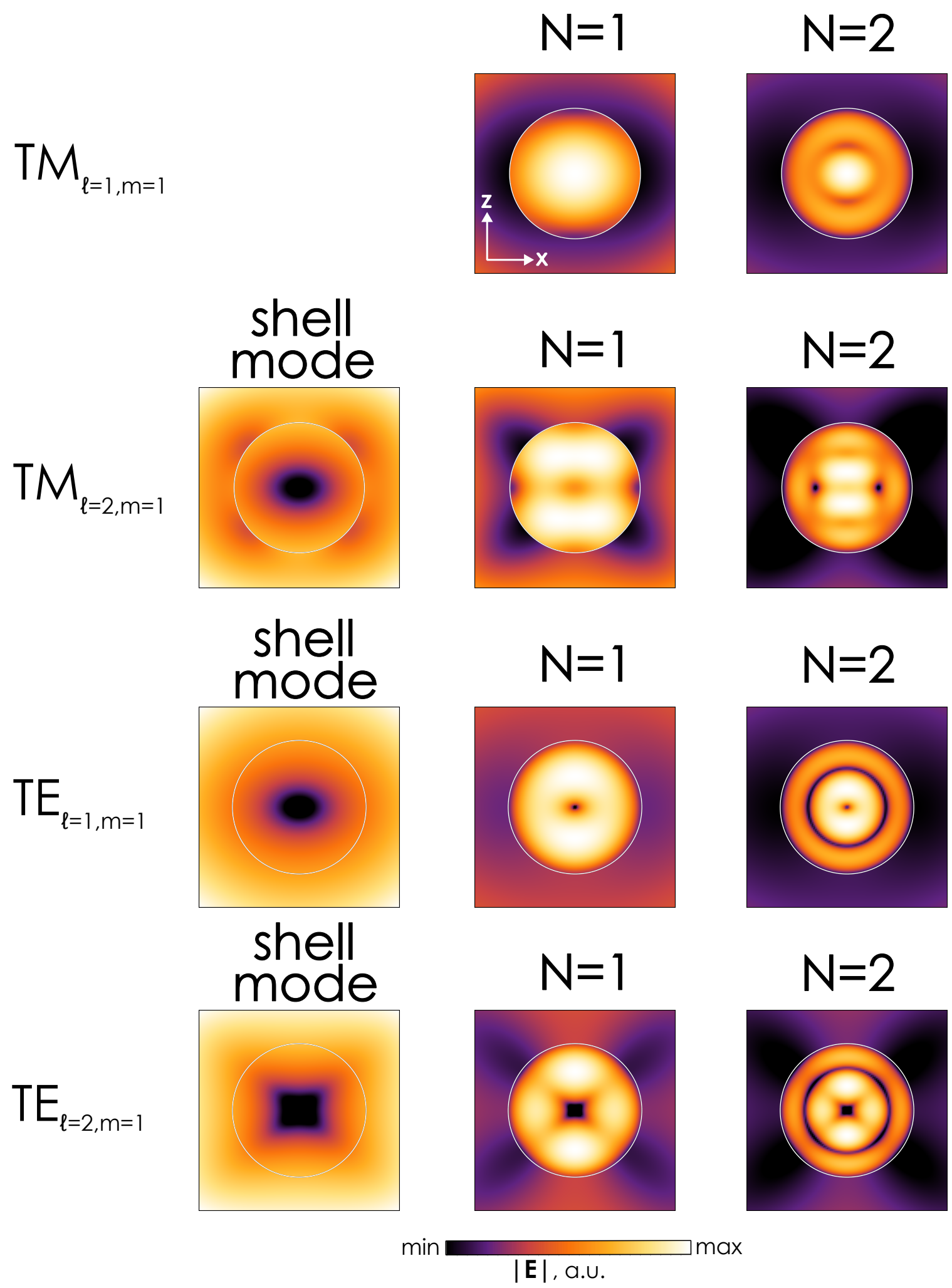

FIG. S1. Electric field profiles of the $TM_{\ell=1,2;m=1}$ and $TE_{\ell=1,2;m=1}$ quasinormal modes of an empty spherical Mie void embedded in a transparent dielectric with $\varepsilon_{bg} = 16$. The pictures demonstrate one of the characteristic features of the *shell modes* – they are mainly localized outside the cavity.

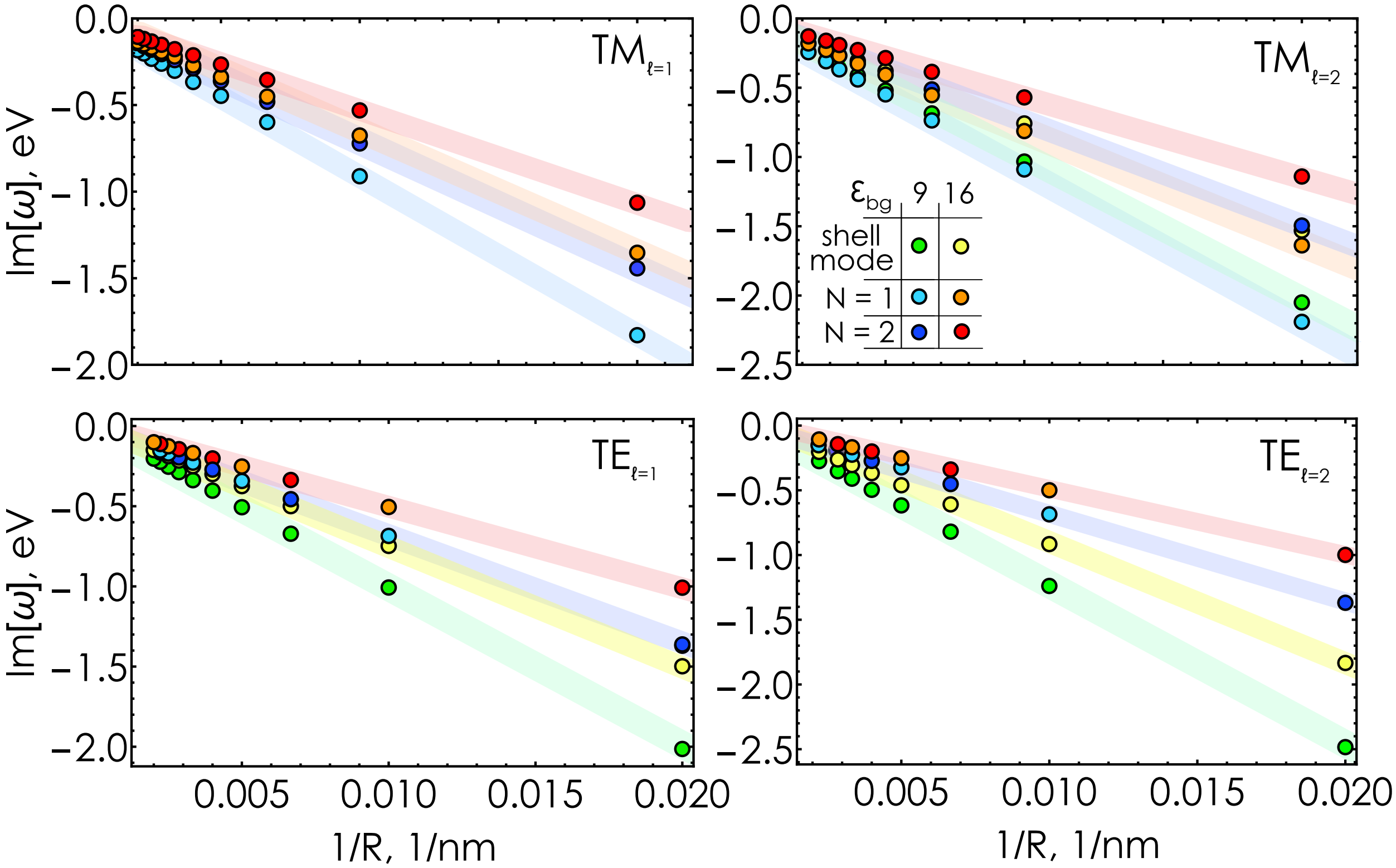

FIG. S2. Imaginary parts of the $TM_{\ell=1,2}$ and $TE_{\ell=1,2}$ QNM frequencies of an empty spherical Mie void surrounded by transparent dielectrics ($\varepsilon_{bg} = 9, 16$) as functions of the void inverse radius. Colored lines are guides for the eye.

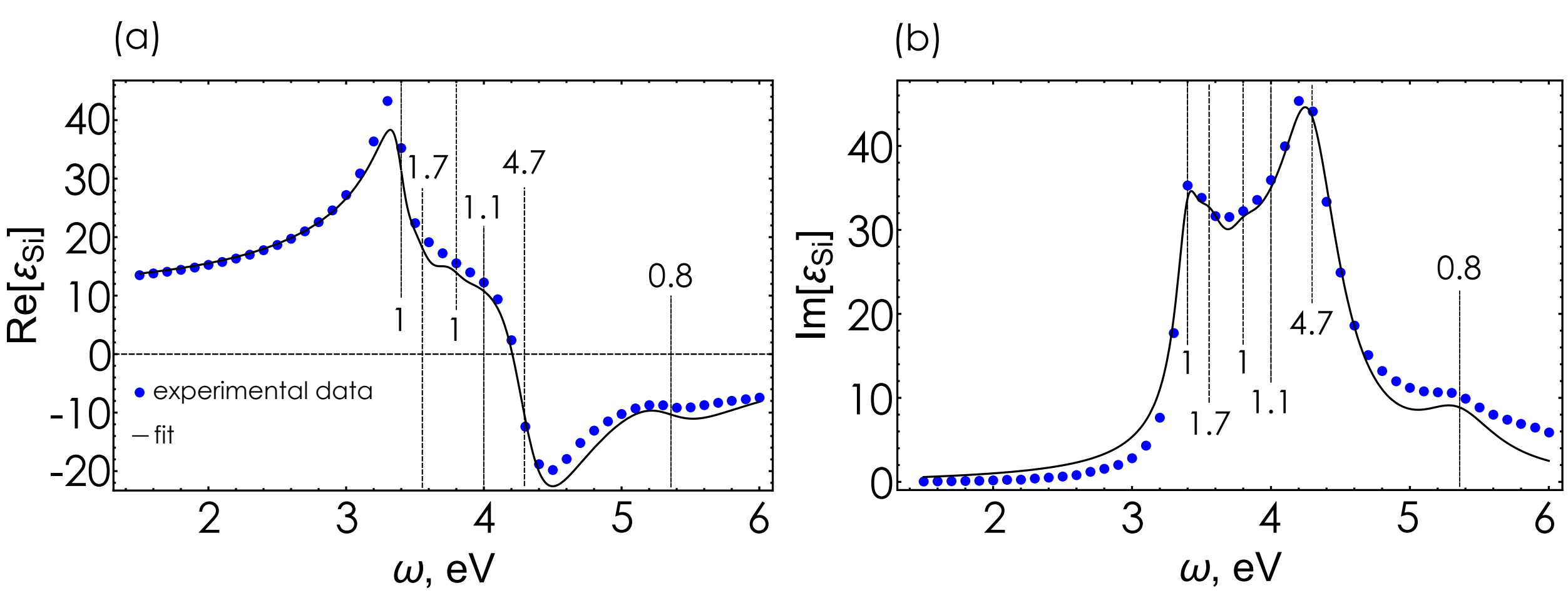

FIG. S3. The fit for the dielectric permittivity of crystalline silicon from [**?**] with a set of Lorentz poles, Eq. (12). The corresponding resonant frequencies and oscillator strengths are marked with black dashed lines and numbers.

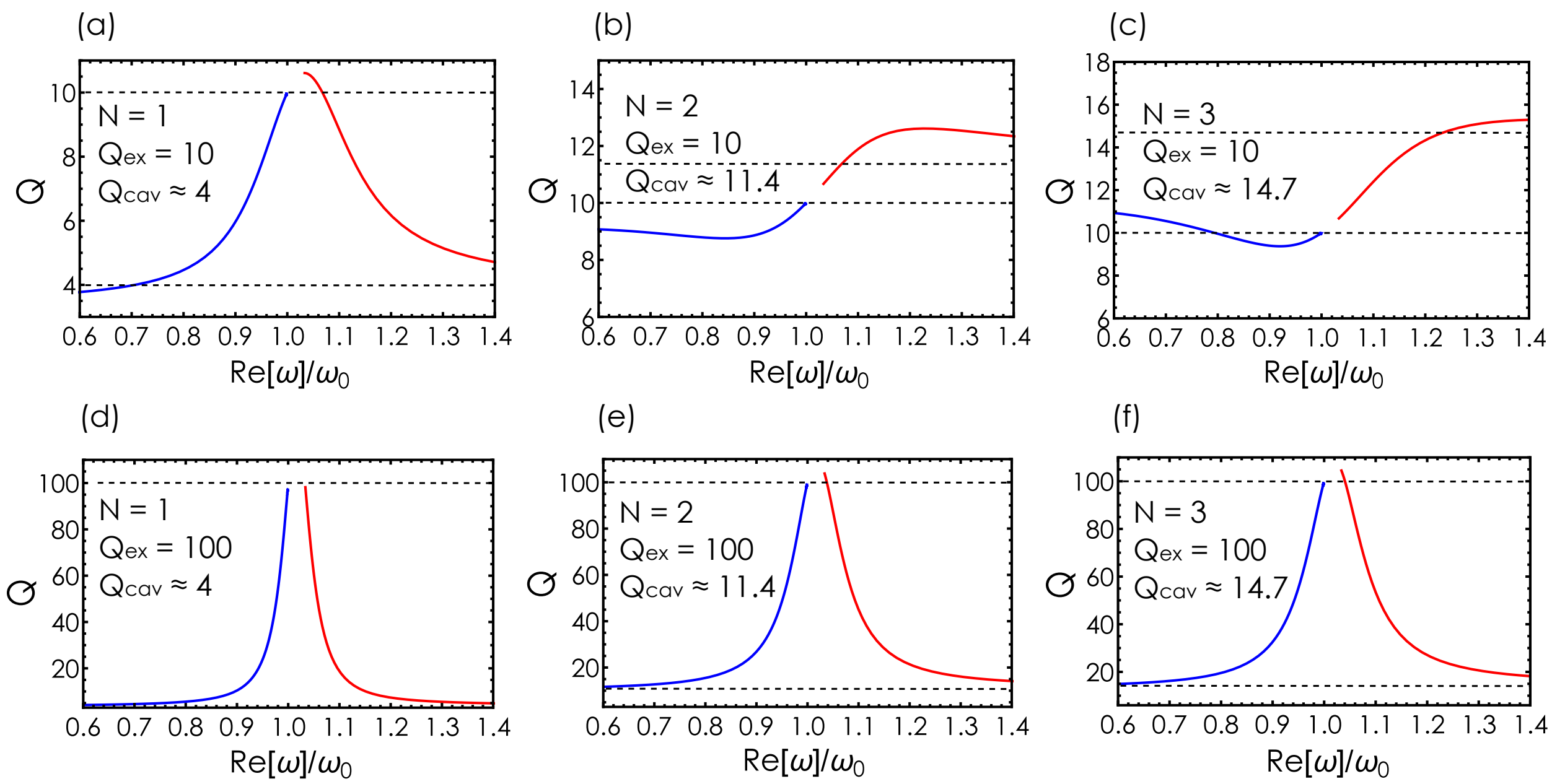

FIG. S4. Q-factors of the upper (red) and lower (blue) polaritons vs. normalized polariton resonant frequencies in the coupled oscillators (2x2 Hamiltonian) model for the $\text{TM}_{\ell=1}$ $N = 1, 2, 3$ modes and background material with $f = 2$, $\varepsilon_\infty = 16$. The coupling strength was estimated as that of a bulk material, $g_{bulk} = \omega_0/2\sqrt{f/(\varepsilon_\infty)}$. The Q-factors of the bare excitonic and photonic components are marked with black dashed lines. The top row corresponds to the low-Q background ($\gamma_v \approx \gamma_{ex}$), the bottom one - to the high-Q background ($\gamma_v \gg \gamma_{ex}$).

TABLE S1. The parameters used for calculating the QNM field profiles, Fig. 10. The values are given with an accuracy of up to three decimal places. For simplicity of illustration, $\omega_0 = 1$ eV and $\gamma = 0.1$ eV for each case, $\varepsilon_\infty = 16$.

	TM				TE			
	Loc.		Deloc.		Loc.		Deloc.	
ℓ	1	2	1	2	1	2	1	2
f	1	2	1	2	1	2	1	2
r (nm)	1200	770	1000	650	870	1150	740	945
$\Re e[\omega]$ (eV)	0.993	0.987	1.206	1.198	0.998	0.981	1.199	1.204
$\|\Im m[\omega]\|$ (eV)	0.029	0.045	0.057	0.143	0.083	0.025	0.073	0.063

S2. RESONANCES AND Q-FACTOR OF MIE VOIDS IN A HIGH-REFRACTIVE-INDEX MEDIA

We consider a spherical cavity (void) of radius R in a dielectric background with permittivity $\varepsilon_{\rm bg}$ (relative permeability $\mu = 1$). The refractive index ratio is

$$\eta = \frac{n_v}{n_{\rm bg}}, \qquad n_{\rm bg} = \sqrt{\varepsilon_{\rm bg}}, \qquad n_v = \sqrt{\varepsilon_{\rm v}}.$$

The complex eigenfrequencies of the Mie resonances are given by the solutions of the following equations:

$$\text{TM}: \quad n_{\rm bg}\xi_\ell(n_{\rm bg}k_0R)\psi'_\ell(n_vk_0R) - n_v\psi_\ell(n_vk_0R)\xi'_\ell(n_{\rm bg}k_0R) = 0, \tag{S1}$$

$$\text{TE}: \quad n_v\xi_\ell(n_{\rm bg}k_0R)\psi'_\ell(n_vk_0R) - n_{\rm bg}\psi_\ell(n_vk_0R)\xi'_\ell(n_{\rm bg}k_0R) = 0. \tag{S2}$$

Here The Riccati–Bessel functions are defined as

$$\psi_\ell(u) = uj_\ell(u), \qquad \xi_\ell(u) = uh_\ell^{(1)}(u), \tag{S3}$$

where $j_\ell(u)$ and $h_\ell^{(1)}(u)$ are the spherical Bessel and Hankel functions.

Let's introduce the dimensionless size parameter $z = n_vk_0R$ and find the solutions of Eqs. (S1) and (S2). In this case η plays the role of a small parameter (limit $\varepsilon_{\rm bg} \to \infty$). Eqs. (S1) and (S2) can be rewritten as

$$\text{TM}: \quad \eta\psi_\ell(z)\xi'_\ell(z/\eta) - \xi_\ell(z/\eta)\psi'_\ell(z) = 0, \tag{S4}$$

$$\text{TE}: \quad \psi_\ell(z)\xi'_\ell(z/\eta) - \eta\xi_\ell(z/\eta)\psi'_\ell(z) = 0. \tag{S5}$$

Utilizing $\xi'_\ell(z/\eta)/\xi_\ell(z/\eta) \to i$ as $z/\eta \to \infty$, one obtains

$$\text{TM}: \quad i\eta\psi_\ell(z) - \psi'_\ell(z) = 0, \tag{S6}$$

$$\text{TE}: \quad i\psi_\ell(z) - \eta\psi'_\ell(z) = 0. \tag{S7}$$

A. TM modes

Let us obtain the corresponding corrections separately for each polarization in the following form

$$z = z_0 + \alpha\eta + \beta\eta^2 \tag{S8}$$

In the case of TM polarization, according to Eq. (S6), z_0 satisfies

$$\psi'_\ell(z_0) = 0 \quad \Leftrightarrow \quad j_\ell(z_0) + z_0 j'_\ell(z_0) = 0. \tag{S9}$$

For the components of Eq. (S6) the Taylor expansion will take the following forms

$$i\eta\psi_\ell(z) = i\eta\psi_\ell(z_0) + i\frac{\psi''_\ell(z_0)}{2}\alpha^2\eta^3 + i\psi''_\ell(z_0)\alpha\beta\eta^4 + ... \tag{S10}$$

$$\psi'_\ell(z) = \psi''_\ell(z_0)\alpha\eta + \psi''_\ell(z_0)\beta\eta^2 + \frac{\psi'''_\ell(z_0)}{2}\alpha^2\eta^2 + ... \tag{S11}$$

The obtained expansions result in the following corrections to z

$$\eta^0: \quad \psi'_\ell(z_0) = 0, \tag{S12}$$

$$\eta^1: \quad i\psi_\ell(z_0) - \psi''_\ell(z_0)\alpha = 0, \tag{S13}$$

$$\eta^2: \quad \psi''_\ell(z_0)\beta + \frac{\psi'''_\ell(z_0)}{2}\alpha^2 = 0. \tag{S14}$$

Therefore,

$$z = z_0 + i\eta\frac{\psi_\ell(z_0)}{\psi''_\ell(z_0)} + \frac{\eta^2}{2}\frac{\psi'''_\ell(z_0)}{\psi''_\ell(z_0)}\left(\frac{\psi_\ell(z_0)}{\psi''_\ell(z_0)}\right)^2 + ... \tag{S15}$$

One can simplify Eq. (S15) by utilizing the standard equations for Riccati-Bessel functions

$$\psi''_\ell + \left(1 - \frac{\ell(\ell+1)}{z^2}\right)\psi_\ell = 0, \tag{S16}$$

$$\frac{\psi_\ell(z_0)}{\psi''_\ell(z_0)} = -\left(1 - \frac{\ell(\ell+1)}{z_0^2}\right)^{-1}, \tag{S17}$$

$$\psi'''_\ell(z_0) + 2\frac{\ell(\ell+1)}{z_0^3}\psi_\ell(z_0) = 0, \tag{S18}$$

$$\frac{\psi'''_\ell(z_0)}{\psi''_\ell(z_0)} + 2\frac{\ell(\ell+1)}{z_0^3}\frac{\psi_\ell(z_0)}{\psi''_\ell(z_0)} = 0, \tag{S19}$$

$$\frac{\psi'''_\ell(z_0)}{\psi''_\ell(z_0)} = \frac{2\frac{\ell(\ell+1)}{z_0^3}}{\left(1 - \frac{\ell(\ell+1)}{z_0^2}\right)}. \tag{S20}$$

Introducing a new parameter for compactness,

$$A = \left(1 - \frac{\ell(\ell+1)}{z_0^2}\right)^{-1}, \tag{S21}$$

We finally obtain

$$z = z_0 - iA\eta + \frac{\ell(\ell+1)}{z_0^3}A^3\eta^2 + O(\eta^3). \tag{S22}$$

B. TE modes

As follows from Eq. (S7), z_0 satisfies

$$\psi_\ell(z_0) = 0 \quad \Leftrightarrow \quad j_\ell(z_0) = 0. \tag{S23}$$

By the same logic as for TM polarization, for Eq. (S7), we obtain

$$z = z_0 + \alpha\eta + \beta\eta^2, \tag{S24}$$

$$i\psi_\ell(z) = i\psi'_\ell(z_0)\alpha\eta + i\psi'_\ell(z_0)\beta\eta^2 + ... \tag{S25}$$

$$\eta\psi'_\ell(z) = \eta\psi'_\ell(z_0) + ... \tag{S26}$$

$$\eta^0 : \quad \psi_\ell(z_0) = 0, \tag{S27}$$

$$\eta^1 : \quad i\psi'_\ell(z_0)\alpha - \psi'_\ell(z_0) = 0, \tag{S28}$$

$$\eta^2 : \quad i\psi'_\ell(z_0)\beta = 0. \tag{S29}$$

Hence,

$$z = z_0 - i\eta + O(\eta^3). \tag{S30}$$

C. Analytical expressions for Q-factors

Let $z = z_0 + \delta z$. Utilizing the obtained first-order corrections, Eqs. (S22) and (S30), we come to the following expressions for δz

$$\text{TE modes:} \qquad \delta z \approx -i\eta, \tag{S31}$$

$$\text{TM modes:} \qquad \delta z \approx -i\eta \, \frac{1}{1 - \ell(\ell+1)/z_0^2}. \tag{S32}$$

One can see that the corrections are purely imaginary. As we defined $Q = \Re e[\omega]/2|\Im m[\omega]| = \Re e[z]/2|\Im m[z]|$, the asymptotics of Q-factors of Mie voids in a high-refractive index medium is given by

$$\begin{aligned} \text{TE modes :} \quad Q &\approx \frac{z_0}{2}\sqrt{\frac{\varepsilon_{\rm bg}}{\varepsilon_v}}, & j_\ell(z_0) = 0, \\ \text{TM modes :} \quad Q &\approx \frac{z_0}{2}\left|1 - \frac{\ell(\ell+1)}{z_0^2}\right|\sqrt{\frac{\varepsilon_{\rm bg}}{\varepsilon_v}}, & z_0 j'_\ell(z_0) + j_\ell(z_0) = 0. \end{aligned} \tag{S33}$$

Thus, in the limit $\varepsilon_{\rm bg} \to \infty$, the spectrum tends to that of a perfectly conducting spherical cavity, and the radiative Q-factors increase as $\sqrt{\varepsilon_{\rm bg}}$.

S3. LOW-Q MODES OF MIE VOIDS

In the limit $\varepsilon_{\rm bg} \to \infty$, Eqs. (S1) and (S2) can be written as:

$$\text{TM}: \quad \psi'_\ell(n_v k_0 R)\xi_\ell(n_{\rm bg} k_0 R) = 0, \tag{S34}$$

$$\text{TE}: \quad \psi_\ell(n_v k_0 R)\xi'_\ell(n_{\rm bg} k_0 R) = 0. \tag{S35}$$

These equations admit two distinct classes of solutions. The first class corresponds to Mie resonances, which are confined within the void owing to the dielectric contrast. An asymptotic expansion for the complex eigenfrequencies of these modes has been derived in the previous section. The second class corresponds to external, strongly delocalized modes, whose complex eigenfrequencies are determined by the following equations:

$$\text{TM:} \quad \xi_\ell(n_{\rm bg} k_0 R) = 0, \tag{S36}$$

$$\text{TE:} \quad \xi'_\ell(n_{\rm bg} k_0 R) = 0. \tag{S37}$$

The lowest complex roots of these equations can be obtained approximately from the expansion of the Riccati-Hankel functions into the asymptotic series:

$$\text{TM } (\ell = 1): \quad \xi_1(z) = -e^{iz}\left(1 + \frac{i}{z}\right), \tag{S38}$$

$$\text{TM } (\ell = 2): \quad \xi_2(z) = ie^{iz}\left(1 + \frac{3i}{z} - \frac{3}{z^2}\right), \tag{S39}$$

$$\text{TE } (\ell = 1): \quad \xi'_1(z) = -e^{iz}\left(i - \frac{1}{z} - \frac{i}{z^2}\right), \tag{S40}$$

$$\text{TE } (\ell = 2): \quad \xi'_2(z) = ie^{iz}\left(i - \frac{3}{z} - \frac{6i}{z^2} + \frac{6}{z^3}\right). \tag{S41}$$

The solutions of these equations are the following:

$$\text{TM } (\ell = 1): \quad z = -i, \tag{S42}$$

$$\text{TM } (\ell = 2): \quad z = \pm\frac{\sqrt{3}}{2} - \frac{3i}{2}, \tag{S43}$$

$$\text{TE } (\ell = 1): \quad z = \pm\frac{\sqrt{3}}{2} - \frac{i}{2}, , \tag{S44}$$

$$\text{TE } (\ell = 2): \quad z \approx \pm 1.81 - i0.70; \quad z \approx -1.60i. \tag{S45}$$

S4. POLARITONIC SPECTRA OF MIE VOIDS FILLED WITH RESONANT MEDIUM

We consider a Mie void filled with resonant medium with dielectric permittivity in the single-pole Lorentz form

$$\varepsilon_v(\omega) = \varepsilon_\infty + f\frac{\omega_0^2}{\omega_0^2 - \omega^2 - i\gamma_{\rm ex}\omega} = \varepsilon_\infty + \delta\varepsilon(\omega), \tag{S46}$$

where ε_∞ is the high-frequency permittivity, f is the reduced oscillator strength, ω_0 and γ_{ex} are the resonance frequency and linewidth, or the resonant frequency and phenomenological decay rate of the exciton.

According to Eqs. (S22) and (S30), the dimensionless parameter z can be expressed as

$$\text{TE:} \qquad n_v(\omega)\frac{\omega R}{c} = z_0 - i\frac{n_v(\omega)}{n_{\rm bg}}, \tag{S47}$$

$$\text{TM:} \qquad n_v(\omega)\frac{\omega R}{c} = z_0 - iA\frac{n_v(\omega)}{n_{\rm bg}}. \tag{S48}$$

In the limit $\delta\varepsilon \to 0$ ($n_v \to n_\infty = \sqrt{\varepsilon_\infty}$), we can write the resonant frequency ($\Re e[\omega]$) of the void

$$\text{TE:} \qquad \omega_v = \frac{z_0 c}{R n_\infty}; \qquad j_\ell(z_0) = 0, \tag{S49}$$

$$\text{TM:} \qquad \omega_v = \frac{z_0 c}{R n_\infty}; \qquad j_\ell(z_0) + z_0 j_\ell'(z_0) = 0. \tag{S50}$$

For a high-index background, the radiative leakage of the void mode gives a cavity loss rate (in terms of energy, $\Im m[\omega] = -\gamma_v/2$)

$$\text{TE}: \quad \gamma_v = \frac{2c}{n_{\rm bg}R}, \tag{S51}$$

$$\text{TM}: \quad \gamma_v = A\frac{2c}{n_{\rm bg}R}. \tag{S52}$$

Near the exciton $\omega_0^2 - \omega^2 \approx 2\omega_0(\omega_0 - \omega)$, which leads to

$$\delta\varepsilon(\omega) = f\frac{\omega_0^2}{\omega_0^2 - \omega^2 - i\gamma_{\rm ex}\omega} \approx \frac{f\omega_0}{2}\frac{1}{\omega_0 - \omega - i\gamma_{\rm ex}/2}. \tag{S53}$$

For the case of $f \ll 1$ the refractive index of a Mie void is

$$n_v(\omega) = n_\infty + \frac{\delta\varepsilon(\omega)}{2n_\infty}. \tag{S54}$$

Combining all together one obtains

$$\text{TE:}\quad n_\infty \frac{\omega R}{c} + i\frac{n_\infty}{n_{\rm bg}} + \frac{\omega R}{c}\frac{1}{2n_\infty}\frac{f\omega_0}{2}\frac{1}{\omega_0-\omega-i\gamma_{\rm ex}/2} = z_0, \quad j_\ell(z_0)=0; \tag{S55}$$

$$\text{TM:}\quad n_\infty \frac{\omega R}{c} + iA\frac{n_\infty}{n_{\rm bg}} + \frac{\omega R}{c}\frac{1}{2n_\infty}\frac{f\omega_0}{2}\frac{1}{\omega_0-\omega-i\gamma_{\rm ex}/2} = z_0, \quad j_\ell(z_0)+z_0 j_\ell'(z_0)=0. \tag{S56}$$

In the vicinity of $\omega_v \approx \omega_0$, both of these equations can be reduced to the same form

$$(\omega-\omega_v+i\gamma_v/2)(\omega-\omega_0+i\gamma_{\rm ex}/2) = \frac{f\omega_0^2}{4n_\infty^2} = g^2. \tag{S57}$$

Therefore, the coupling constant is

$$g = \frac{\omega_0}{2}\sqrt{\frac{f}{\varepsilon_\infty}}. \tag{S58}$$
